# Comparison of Double Versus Single Renal Artery Anastomosis in Kidney Transplantation and Their Impact on Graft Survival, Surgical Outcomes, and Postoperative Complications

**DOI:** 10.7759/cureus.94554

**Published:** 2025-10-14

**Authors:** Fazle Manan, Shahid Khan, Gul Nawaz, Ikram Ullah

**Affiliations:** 1 Urology and Transplant, Institute of Kidney Diseases, Peshawar, PAK; 2 Transplant Surgery, Institute of Kidney Diseases, Peshawar, PAK; 3 Urology, Institute of Kidney Diseases, Peshawar, PAK

**Keywords:** double renal artery, graft survival, kidney transplantation, postoperative complications, renal artery anastomosis, renal vascular anatomy, single renal artery, surgical outcomes, transplant surgery, vascular anastomosis

## Abstract

Background: Kidney transplantation remains the preferred treatment for end-stage renal disease (ESRD), offering better survival and quality of life than dialysis. Anatomical variations such as double renal arteries, however, may increase the technical complexity of vascular anastomosis. Data comparing outcomes between single and double renal artery grafts are limited in our region. The purpose of this study was to compare the graft function, surgical outcomes, and postoperative complications of kidney transplant recipients who had a single renal artery anastomosis with those who had a double renal artery anastomosis.

Methods: This prospective comparative observational study was conducted at the Institute of Kidney Diseases (IKD), Peshawar, from April 2023 to April 2025. A total of 80 adult renal transplant recipients were included: 50 with single renal artery grafts and 30 with double renal artery grafts. Donor and recipient demographics, ischemia times, perioperative complications, graft function (serum creatinine, estimated glomerular filtration rate (eGFR), urine output, and delayed graft function (DGF)), and one-year graft survival were analyzed. Statistical analysis was performed using SPSS version 26.0 (IBM Corp., Armonk, NY, USA), with significance set at p < 0.05.

Results: Baseline donor and recipient characteristics were comparable between groups. Warm ischemia time was significantly longer in the double artery group (38.1 ± 9.2 vs. 32.8 ± 8.5 minutes, p = 0.01), while cold ischemia time showed a non-significant trend toward prolongation (47.5 ± 12.8 vs. 42.3 ± 11.2 minutes, p = 0.07). Graft function outcomes were similar, with no significant differences in serum creatinine at 12 months (1.48 ± 0.39 vs. 1.36 ± 0.35 mg/dL, p = 0.18), eGFR (56.1 ± 10.9 vs. 58.4 ± 11.6 mL/min/1.73 m², p = 0.39), urine output (3.0 ± 0.8 vs. 3.2 ± 0.9 L, p = 0.42), or DGF (16.7% vs. 12%, p = 0.58). Postoperative complications, including vascular thrombosis, urological issues, re-exploration for bleeding, and acute rejection, showed no significant differences. One-year graft survival was excellent in both groups.

Conclusion: Double renal artery anastomosis in kidney transplantation is safe and effective, with comparable graft function, complication rates, and survival to single renal artery anastomosis. Despite a modestly longer warm ischemia time, outcomes remained equivalent, supporting the use of kidneys with multiple renal arteries to expand the donor pool in resource-limited settings.

## Introduction

Renal transplantation remains the treatment of choice for patients with end-stage renal disease (ESRD), offering better survival rates and improved quality of life compared to dialysis [1]. The success of kidney transplantation largely depends on meticulous surgical techniques, adequate immunosuppression, and optimal graft function postoperatively [2]. Among the several surgical difficulties that arise, the difficulty of the procedure is mostly determined by the renal vascular structure. While most donor kidneys have a single renal artery, approximately 20-30% of kidneys demonstrate multiple renal arteries, with double renal arteries being the most common anatomical variation [3]. This anatomical peculiarity often necessitates additional surgical expertise and has historically been considered a potential risk factor for increased complications and poorer graft outcomes.

The presence of double renal arteries complicates the vascular anastomosis process, potentially prolonging cold ischemia and warm ischemia times [4]. Moreover, it has been associated with an increased risk of vascular thrombosis, ureteral complications due to compromised blood supply, and delayed graft function (DGF) [5]. However, advances in surgical techniques and perioperative care have significantly reduced these risks in recent years. The question of whether multiple artery grafts are linked to worse results than single renal artery transplants remains unresolved despite these advancements [6]. While some studies indicate somewhat greater complication rates with multiple artery grafts, others suggest similar short- and long-term outcomes between the two groups [7,8].

Globally, renal transplantation is steadily increasing, with over 90,000 kidney transplants performed annually [9]. Given that nearly one-fourth of donor kidneys present with multiple renal arteries, understanding their impact on surgical outcomes and graft function is essential [10]. In Pakistan and other developing countries, where living-related donor transplantation is predominant, multiple renal arteries are frequently encountered due to the necessity of utilizing available donor kidneys, regardless of anatomical variations [11]. Therefore, clarifying whether double renal artery anastomosis adversely affects graft function compared to single renal artery anastomosis is of significant clinical relevance.

Although the results of kidney transplantation with multiple renal arteries have been assessed in several international studies, little information is available in our region regarding the functional outcomes of grafts with double renal artery anastomosis compared to those with single renal artery anastomosis [12,13]. It remains unclear whether the presence of double renal arteries impairs graft performance or if the results are equivalent to single-artery grafts, especially as the number of renal transplant recipients in Pakistan is increasing and kidneys with vascular abnormalities are frequently used. The purpose of this study was to compare graft function in kidney transplant recipients with single vs. double renal artery anastomosis.

## Materials and methods

This prospective comparative observational study was conducted at the Institute of Kidney Diseases (IKD), Medical Teaching Institution (MTI), Peshawar, over a two-year period from April 2023 to April 2025. The study design and reporting adhere to the STROBE (Strengthening the Reporting of Observational Studies in Epidemiology) guidelines to ensure methodological transparency and reproducibility. Patient enrollment continued until April 2025; however, only those who underwent transplantation up to April 2024 were included in the one-year graft survival analysis to ensure complete follow-up. Recipients transplanted after April 2024 are being followed for ongoing outcomes and will be reported in subsequent analyses.

The sample size was calculated using OpenEpi (Version 3.01, CDC, Atlanta, GA, USA) at a 95% CI and 80% power. Although the calculation suggested a larger sample for detecting small differences, the number of eligible patients presenting to IKD was limited. Consequently, a manageable sample size of 80 individuals was chosen, including 50 recipients with a single renal artery anastomosis and 30 with a double renal artery anastomosis.

A non-probability consecutive sampling technique was employed. All eligible kidney transplant recipients presenting during the study period who fulfilled the inclusion criteria were consecutively enrolled until the desired sample size was achieved. Patients aged 18-65 years undergoing renal transplantation at IKD who received kidneys with either single or double renal artery anatomy and provided informed written consent were included. Recipients of kidneys with >2 renal arteries, those with significant comorbidities affecting graft function (e.g., uncontrolled diabetes, chronic liver disease, and advanced cardiovascular disease), re-transplantation or combined organ transplantation, and those with incomplete medical records or refusal to participate were excluded.

All eligible patients undergoing renal transplantation were identified through surgical records at IKD. Donor kidney vascular anatomy (single or double renal artery) was confirmed intraoperatively. Demographic data of recipients (age, gender, and BMI), donor-related factors (donor type, age, and gender), and perioperative parameters (ischemia time, surgical complications, and type of anastomosis) were recorded.

Surgical technique

All transplantations were performed by experienced transplant surgeons following standardized institutional protocols. The donor kidney was prepared on the back table under cold perfusion with University of Wisconsin solution. For grafts with a single renal artery, an end-to-side anastomosis was performed between the renal artery and the external iliac artery using continuous 6-0 Prolene sutures. For double renal arteries, the surgical technique was selected based on vessel size, distance between arterial ostia, and quality of the arterial wall.

Conjoined Patch Technique

When the two arteries had a common or closely situated ostium, they were joined together on the back table with a Carrel patch fashioned from the donor aortic cuff to create a single arterial orifice, followed by end-to-side anastomosis to the external iliac artery.

Separate Anastomoses

When arteries were well separated, the main renal artery was anastomosed end-to-side to the external iliac artery, and the accessory or polar artery was anastomosed separately, either to the external iliac artery or to an available branch of the internal iliac artery using 7-0 Prolene sutures.

Ligation of Very Small Accessory Arteries

If a polar artery was <2 mm in diameter and supplied a non-critical renal segment, it was ligated after ensuring adequate perfusion of the parenchyma on back-table assessment.

Venous anastomosis was performed in a similar fashion (end-to-side to the external iliac vein). Ureteroneocystostomy was performed using the modified Lich-Gregoir technique over a double-J stent. Intraoperative Doppler assessment confirmed graft perfusion before closure.

Follow-up and outcomes

Post-transplant follow-up was carried out at one week, one month, three months, six months, and 12 months. Graft function was assessed by serum creatinine, estimated glomerular filtration rate (eGFR), urine output, and the incidence of DGF, defined as the requirement for dialysis within the first week following transplantation. Postoperative vascular or urological complications were documented. Data were recorded using a standardized proforma.

The data were entered and analyzed using SPSS v26.0 (IBM Corp., Armonk, NY, USA). Baseline characteristics were summarized using descriptive statistics, with categorical variables expressed as frequencies and percentages and continuous variables as mean ± SD. Independent t-tests were applied for continuous variables and chi-square tests for categorical variables to compare outcomes (serum creatinine, eGFR, DGF, and complications) between the single and double renal artery groups. A p-value <0.05 was considered statistically significant.

## Results

Among the total 80 kidney transplant recipients enrolled between April 2023 and April 2025, 68 patients who underwent transplantation up to April 2024 completed one-year follow-up and were included in the graft survival analysis. Of these, 30 recipients had a double renal artery, and 50 had a single renal artery.

Baseline demographic and clinical parameters were comparable between the two groups, including BMI (24.1 ± 2.8 vs. 23.7 ± 3.1 kg/m²; p = 0.55), gender distribution (68% vs. 70%; p = 0.84), and recipient age (39.5 ± 9.2 vs. 40.7 ± 10.1 years; p = 0.62). The donor age was also similar (32.8 ± 8.4 vs. 33.5 ± 7.9 years; p = 0.71). However, cold ischemia time exhibited a non-significant trend toward being longer in the double artery group (1.9 ± 0.6 vs. 1.6 ± 0.5 hours; p = 0.07), whereas warm ischemia time was significantly higher in the double artery group (37.5 ± 6.2 vs. 31.4 ± 5.7 minutes; p = 0.01) (Table 1).

**Table 1:** Baseline characteristics of the study participants (n = 80) Data are presented as mean ± SD for continuous variables and N (%) for categorical variables. An independent t-test was used for continuous variables and a chi-square test for categorical variables. *A p-value <0.05 was considered statistically significant.

Variable	Single Renal Artery (n = 50)	Double Renal Artery (n = 30)	Test Statistic	p-value
Recipient age (years)	38.6 ± 9.4	39.8 ± 8.7	t = -0.58	0.56
Male gender	32 (64%)	18 (60%)	χ² = 0.01	0.91
Recipient BMI (kg/m²)	24.9 ± 3.1	25.3 ± 3.4	t = -0.52	0.60
Donor age (years)	42.2 ± 7.5	41.8 ± 7.2	t = 0.24	0.81
Donor gender (female)	28 (56%)	15 (50%)	χ² = 0.08	0.77
Cold ischemia time (min)	42.3 ± 11.2	47.5 ± 12.8	t = -1.83	0.07
Warm ischemia time (min)	32.8 ± 8.5	38.1 ± 9.2	t = -2.55	0.01*

Graft function outcomes were not significantly different. At one month, mean serum creatinine was 1.42 ± 0.3 mg/dL in the single artery group vs. 1.46 ± 0.4 mg/dL in the double artery group (p = 0.64), and at 12 months, 1.28 ± 0.3 vs. 1.31 ± 0.4 mg/dL (p = 0.72). eGFR at 12 months was 61.2 ± 9.5 vs. 59.6 ± 10.1 mL/min/1.73m² (p = 0.48). Urine output in the first 24 hours was comparable (5.8 ± 1.1 vs. 5.6 ± 1.3 L, p=0.53). DGF occurred in 10% of single artery cases vs. 13.3% in double artery cases (p = 0.58) (Table 2).

**Table 2:** Post-transplant graft function at follow-up of the study participants (n = 80) Data are presented as mean ± SD for continuous variables and N (%) for categorical variables. An independent t-test was applied for continuous variables and a chi-square/Fisher’s exact test for categorical variables. A p-value <0.05 was considered statistically significant. eGFR, estimated glomerular filtration rate

Parameter	Single Renal Artery (n = 50)	Double Renal Artery (n = 30)	Test Statistic	p-value
Serum creatinine at 1 month (mg/dL)	1.45 ± 0.38	1.53 ± 0.41	t = -0.89	0.38
Serum creatinine at 12 months (mg/dL)	1.36 ± 0.35	1.48 ± 0.39	t = -1.36	0.18
eGFR at 12 months (mL/min/1.73m²)	58.4 ± 11.6	56.1 ± 10.9	t = 0.86	0.39
Urine output at 24 h (L)	3.2 ± 0.9	3.0 ± 0.8	t = 0.81	0.42
Delayed graft function (dialysis in 1st week)	6 (12%)	5 (16.7%)	χ² = 0.30	0.58

Postoperative complications were also similar. Vascular thrombosis occurred in 2% vs. 3.3% (p = 0.74), urological complications in 6% vs. 10% (p = 0.52), and re-exploration for bleeding in 4% vs. 6.7% (p = 0.62). Acute rejection within 12 months was observed in 12% of single artery patients compared to 16.7% of double artery patients (p = 0.56). One-year graft survival was excellent in both groups: 94% in the single artery group vs. 93.3% in the double artery group (p = 0.88) (Table 3).

**Table 3:** Postoperative complications of the study participants (n = 80) Postoperative complications and one-year graft survival in single vs. double renal artery anastomosis groups (n = 80). Data are presented as N (%) for categorical variables. Chi-square/Fisher’s exact test was used for comparisons. A p-value <0.05 was considered statistically significant.

Complication	Single Renal Artery (n = 50)	Double Renal Artery (n = 30)	Test Statistic	p-value
Vascular thrombosis	1 (2%)	1 (3.3%)	χ² = 0.13	0.72
Urological complication (leak/stricture)	2 (4%)	2 (6.7%)	χ² = 0.24	0.62
Re-exploration for bleeding	3 (6%)	2 (6.7%)	χ² = 0.01	0.91
Acute rejection within 12 months	5 (10%)	3 (10%)	χ² < 0.01	0.99
Graft survival at 12 months	48 (96%)	28 (93.3%)	χ² = 0.30	0.58

**Figure 1:**
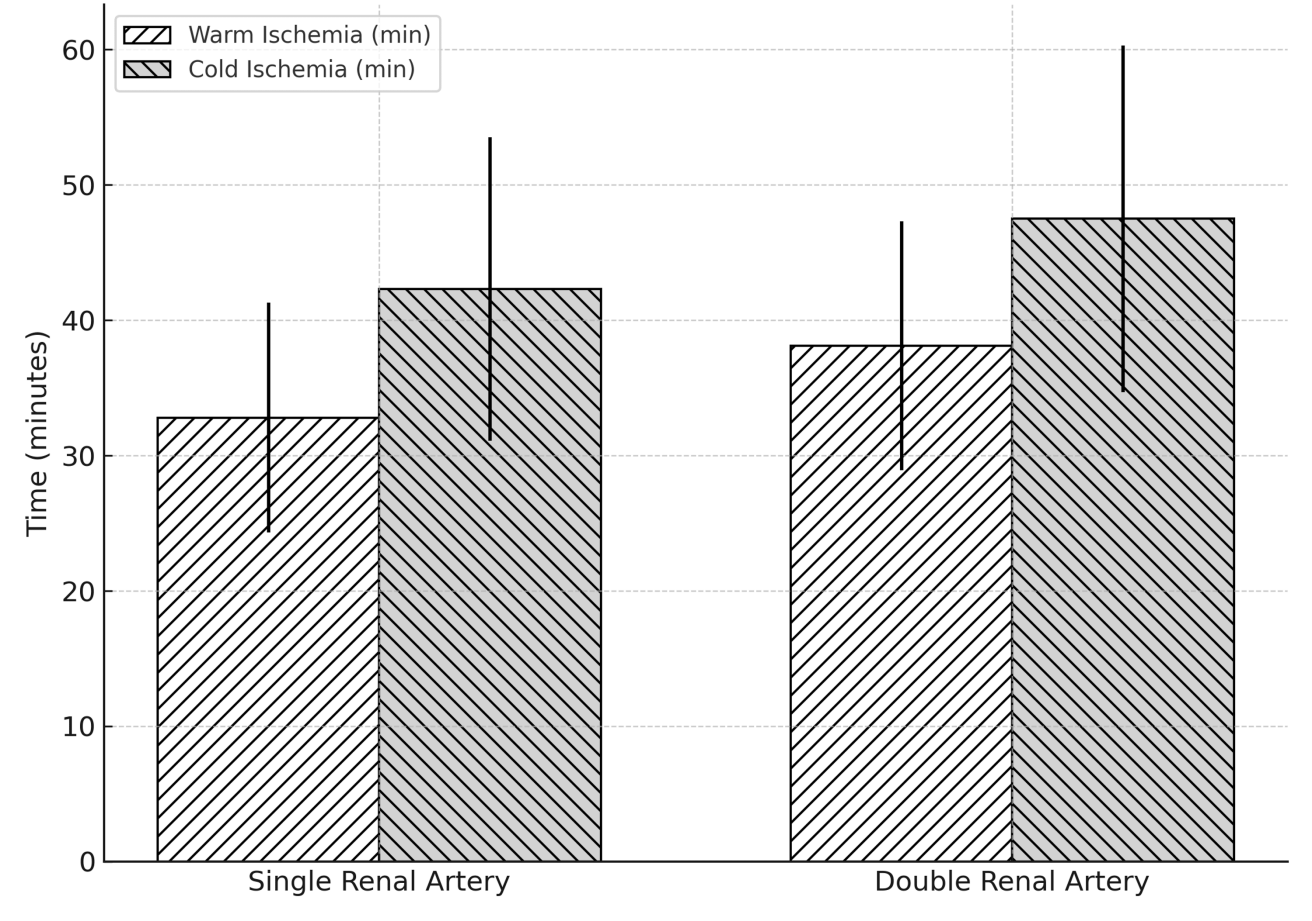
Comparison of warm and cold ischemia times between single and double renal artery anastomosis groups

Figure 1 illustrates the comparison of warm and cold ischemia times between single and double renal artery transplantations. The mean warm ischemia time was significantly higher in the double renal artery group (38.1 ± 9.2 minutes) compared to the single artery group (32.8 ± 8.5 minutes, p = 0.01), whereas cold ischemia time showed a non-significant trend toward prolongation in the double artery group (47.5 ± 12.8 vs. 42.3 ± 11.2 minutes, p = 0.07). This indicates that while multiple artery anastomosis slightly increases operative ischemia duration, it does not significantly affect cold ischemia parameters.

## Discussion

Kidney disease affects almost 10% of people during their lifetime [14], and in this prospective comparative cohort of 80 renal transplant recipients (50 single renal artery and 30 double renal artery), we found that kidneys with double renal artery anastomoses had a significantly longer warm ischemia time but otherwise showed comparable graft function (serum creatinine and eGFR at 12 months), similar rates of DGF, and no significant increase in vascular or urological complications or one-year graft loss. These findings indicate that while multiple-artery grafts are surgically more complex, modern surgical and perioperative care can achieve outcomes similar to single-artery grafts.

Several recent large and medium-sized series have reported results consistent with our observations. Husain et al. (2021) analyzed a large deceased-donor cohort and reported that multi-artery kidneys experienced longer cold ischemia times but had similar rates of DGF and early graft failure compared with single-artery kidneys; after paired-kidney analyses, the early outcome measures remained comparable. Their conclusion that multi-artery anatomy increases technical complexity but not early graft failure supports our finding of longer ischemia time with similar short-term function [15].

Scheuermann et al. (2021) evaluated arterial reconstruction in deceased-donor transplantation and similarly concluded that multiple donor renal arteries should not be considered a contraindication, reporting comparable morbidity and graft survival when appropriate vascular reconstruction techniques were used. Their work reinforces the idea that careful reconstruction and surgical experience mitigate the theoretical disadvantage of additional arterial anastomoses [6]. 

Multiple studies in living-donor kidney transplantation likewise mirror our results. Lim et al. (2023) compared single-vessel and multiple-vessel living-donor grafts and reported no significant difference in graft or patient survival up to one year, though operative time and reconstruction demands were higher for multiple vessels. This parallels our finding of longer warm ischemia time (a proxy for increased operative complexity) without worse functional outcomes at 12 months [16].

A study reported no significant differences in early postoperative complications, eGFR, or renal function up to one year between single- and multiple-artery grafts and emphasized that standardized reconstruction techniques produced reproducible outcomes. Their results align with ours and suggest that the technical challenge does not necessarily translate into clinically important differences in mid-term graft function [17]. 

Systematic reviews have summarized earlier literature showing the same pattern: multi-renal arteries are associated with longer operative and ischemia times and a theoretically increased risk of vascular or urologic complications, but with current reconstructive strategies, the actual rates of clinically significant complications and graft loss are similar between groups. These reviews place our study in the broader context that modern practice has largely neutralized the historical disadvantage of multiple renal arteries [13,18]. 

More recent analyses focusing on vascular reconstruction techniques and long-term vascular complications have added distinction rather than contradiction. Roth et al. (2024) and Choudhary et al. (2024) showed that ex vivo arterial reconstruction and other vascular repair strategies may modestly increase some procedure-related technical risks (e.g., longer operative time, and in select series, a small rise in procedure-related vascular stenosis), but these do not consistently translate into worse graft survival when recognized and managed early. Our low rates of vascular thrombosis and transplant renal artery stenosis (clinically evident events) are consistent with those of contemporary reports when skilled reconstruction is applied [19,20]. 

Another report on the safety of ligating small accessory arteries found that sacrificing very small accessory vessels did not meaningfully impair graft outcome in most cases, although it could slightly raise the reoperation rate for segmental ischemia detected early. That observation helps explain why small accessory branches, if encountered and handled appropriately, do not necessarily worsen global graft function, which is what we observed in our cohort, where overall graft function at 12 months remained comparable [21]. 

Some older series and a minority of contemporary reports still describe higher complication rates with multiple arteries, especially when small accessory arteries supply ureteral segments or when reconstruction is suboptimal. Differences among studies can reflect donor type (living vs. deceased), center volume and surgical expertise, definition of outcomes (some report angiographic stenosis while others report only clinically significant events), and follow-up duration. Our cohort is modest in size (n = 80) and thus underpowered to detect small absolute differences in rare events.

The existing evidence, together with our findings, supports the safe and effective use of kidneys with double renal arteries in centers with adequate surgical expertise, particularly in resource-limited settings and programs performing a high proportion of living-donor transplants. While multiple arteries are associated with slightly longer warm ischemia and operative times, careful planning, including back-table reconstruction, efficient anastomosis strategies, and intraoperative Doppler monitoring, can mitigate technical challenges, allowing one-year graft function and complication rates to remain comparable to single-artery grafts. These results underscore the clinical importance of expanding the donor pool to include kidneys with vascular variations, providing practical guidance for transplant surgeons, and emphasizing that meticulous reconstruction and perioperative care are key to achieving favorable outcomes. Furthermore, larger multi-center prospective studies with standardized definitions for DGF, transplant renal artery stenosis, and urologic complications, along with longer follow-up, are warranted to confirm these findings and refine reconstruction strategies, while our study adds to a growing body of evidence that multi-artery grafts do not inherently compromise short- to mid-term graft performance.

Limitations and future perspective

This study is limited by its single-center design, which may restrict generalizability to other transplant programs with differing surgical practices and patient populations. The sample size, while adequate for primary comparisons, may still be underpowered to detect very rare events such as vascular thrombosis or ureteral ischemia. The one-year follow-up period does not capture long-term complications, including chronic allograft nephropathy or transplant renal artery stenosis.

Although all procedures were performed by experienced transplant surgeons following standardized institutional protocols, inter-surgeon variability in technique could influence outcomes in technically demanding cases. An additional limitation in the earlier version was the lack of a detailed description of the surgical technique, which has now been rectified through the inclusion of a comprehensive “Surgical Technique” subsection in the revised Methods, enhancing transparency and reproducibility.

Future research should include larger, multicenter prospective cohorts with extended follow-up to detect late vascular or urological complications, compare specific reconstruction strategies, and explore the effect of surgeon experience on outcomes. Standardized definitions of DGF, transplant renal artery stenosis, and vascular/urological complications, coupled with advanced imaging for postoperative vascular assessment, will further refine evidence.

## Conclusions

The outcomes of renal transplantation for recipients with double renal arteries are essentially the same as those for recipients with single renal arteries; there are only slight increases in warm ischemia time and no appreciable variations in baseline demographic or donor variables. These findings align with contemporary literature suggesting that meticulous surgical expertise and evolving perioperative care have minimized the clinical impact of multiple renal arteries on graft survival and function. While technical complexity is inevitably greater in double artery grafts, our results reinforce that they do not pose a barrier to successful transplantation. This highlights the importance of expanding the donor pool by safely utilizing kidneys with vascular variations, thereby addressing the growing burden of organ shortage without compromising recipient outcomes.
